# Discovery of photosynthesis genes through whole-genome sequencing of acetate-requiring mutants of *Chlamydomonas reinhardtii*

**DOI:** 10.1371/journal.pgen.1009725

**Published:** 2021-09-07

**Authors:** Setsuko Wakao, Patrick M. Shih, Katharine Guan, Wendy Schackwitz, Joshua Ye, Dhruv Patel, Robert M. Shih, Rachel M. Dent, Mansi Chovatia, Aditi Sharma, Joel Martin, Chia-Lin Wei, Krishna K. Niyogi

**Affiliations:** 1 Division of Molecular Biophysics and Integrated Bioimaging, Lawrence Berkeley National Laboratory, Berkeley, California, United States of America; 2 Department of Plant and Microbial Biology, University of California, Berkeley, California, United States of America; 3 Division of Environmental Genomics and Systems Biology, Lawrence Berkeley National Laboratory, Berkeley, California, United States of America; 4 Feedstocks Division, Joint BioEnergy Institute, Emeryville, California, United States of America; 5 Innovative Genomics Institute, University of California, Berkeley, California, United States of America; 6 Howard Hughes Medical Institute, University of California, Berkeley, California, United States of America; 7 Joint Genome Institute, Berkeley, California, United States of America; TU Kaiserslautern: Technische Universitat Kaiserslautern, GERMANY

## Abstract

Large-scale mutant libraries have been indispensable for genetic studies, and the development of next-generation genome sequencing technologies has greatly advanced efforts to analyze mutants. In this work, we sequenced the genomes of 660 *Chlamydomonas reinhardtii* acetate-requiring mutants, part of a larger photosynthesis mutant collection previously generated by insertional mutagenesis with a linearized plasmid. We identified 554 insertion events from 509 mutants by mapping the plasmid insertion sites through paired-end sequences, in which one end aligned to the plasmid and the other to a chromosomal location. Nearly all (96%) of the events were associated with deletions, duplications, or more complex rearrangements of genomic DNA at the sites of plasmid insertion, and together with deletions that were unassociated with a plasmid insertion, 1470 genes were identified to be affected. Functional annotations of these genes were enriched in those related to photosynthesis, signaling, and tetrapyrrole synthesis as would be expected from a library enriched for photosynthesis mutants. Systematic manual analysis of the disrupted genes for each mutant generated a list of 253 higher-confidence candidate photosynthesis genes, and we experimentally validated two genes that are essential for photoautotrophic growth, *CrLPA3* and *CrPSBP4*. The inventory of candidate genes includes 53 genes from a phylogenomically defined set of conserved genes in green algae and plants. Altogether, 70 candidate genes encode proteins with previously characterized functions in photosynthesis in *Chlamydomonas*, land plants, and/or cyanobacteria; 14 genes encode proteins previously shown to have functions unrelated to photosynthesis. Among the remaining 169 uncharacterized genes, 38 genes encode proteins without any functional annotation, signifying that our results connect a function related to photosynthesis to these previously unknown proteins. This mutant library, with genome sequences that reveal the molecular extent of the chromosomal lesions and resulting higher-confidence candidate genes, will aid in advancing gene discovery and protein functional analysis in photosynthesis.

## Author summary

Oxygenic photosynthesis in plants, algae, and cyanobacteria is responsible for the production of organic matter and oxygen that supports most life on Earth, yet we still do not fully understand all the genes that are necessary for this fundamental biological process. Forward genetics is an unbiased approach to finding genes that are important for a biological process of interest. To identify genes that are necessary for oxygenic photosynthesis in the unicellular green alga *Chlamydomonas reinhardtii*, we sequenced the genomes of 660 mutants that are defective in photosynthesis. By manual curation of each mutant and its disrupted gene(s) and cross-comparison with previous photosynthesis mutant studies, we have identified 253 genes in 328 mutants that are strong candidate genes for having roles in photosynthesis. The list of 253 genes consists of 70 known photosynthesis genes, 169 previously uncharacterized genes, and 14 genes previously characterized for functions unrelated to photosynthesis. In addition, we gained insight into the molecular events that accompany insertional mutagenesis in *Chlamydomonas*, such as large deletions and chromosomal rearrangements. Our mutant library with documented mutant phenotypes, sequenced genomes, and candidate genes will aid in the discovery of photosynthesis genes.

## Introduction

Since the dawn of modern genetics, mutagenesis has been the primary vehicle to perturb the underlying genetic code of organisms, enabling scientists to investigate the genetic determinants underpinning biological systems. In the case of photosynthesis, much has been learned through mutagenesis of the unicellular green alga, *Chlamydomonas reinhardtii*, which has proven to be an indispensable reference organism for investigating the molecular components, regulation, and overall processes of photosynthesis [1,2]. *Chlamydomonas* has a haploid genome and an ability to use acetate as a sole carbon source, which facilitates the isolation and analysis of knock-out mutants that are defective in photosynthesis [3]. Moreover, the advantage of working with a unicellular alga rather than a whole plant has facilitated the speed with which molecular and genetic studies can be carried out [4]. Thus, the development of resources and tools to increase the breadth and depth of genetic studies in *Chlamydomonas* has advanced our ability to understand the molecular basis of photosynthesis.

Numerous large-scale mutagenesis and screening experiments have been carried out in *Chlamydomonas*, with some of the earliest efforts described over half a century ago [3,5,6]. Classical mutagenesis studies have utilized chemical and physical mutagens, which induce untargeted genomic lesions and rearrangements across the genome. Identifying the causative mutations requires genetic mapping through crosses, an approach that is robust but time consuming. Insertional mutagenesis approaches, in which a selectable marker is transformed and randomly integrated into the genome, have facilitated molecular analysis, and many PCR-based techniques have been successfully employed in *Chlamydomomas* to rapidly identify flanking sequence tags (FSTs) from the site of marker insertion [7–14]. However, the efficiency of FST recovery can be low [7] because of the complexity of events accompanying plasmid insertion such as concatemerization, chromosomal deletion or rearrangement, loss of the primer annealing sites, as well as difficulties with PCR from the *Chlamydomonas* nuclear genome, which is GC-rich and contains a high degree of repetitive sequences [15]. High-throughput FST recovery has been achieved in *Chlamydomonas* [8,10] and has offered a large collection of insertional mutants for the scientific community while enabling large-scale mutant analysis of photoautotrophic growth [9].

The advent of next-generation sequencing methods has dramatically improved our ability to identify mutations by whole-genome sequencing (WGS). In *Chlamydomonas*, this approach was initially combined with linkage mapping to identify point mutations in flagellar mutants [11,12], and it was used subsequently for point mutations affecting the cell cycle [13,14] and light signaling [16,17]. In the case of insertional mutants, WGS has been used extensively to identify insertion sites in bacteria and some microbial eukaryotes with smaller genomes [18–20] but only for a relatively small number of mutants in *Chlamydomonas* [21]. In maize, due to its large genome, high-throughput next-generation sequencing of *Mu* transposon insertion sites has been applied only after enrichment for the transposon sequence [22], whereas the large volume of insertion site information of T-DNA insertion lines in *Arabidopsis* was obtained from traditional PCR-based FST isolation [23–25].

We have previously generated a large insertional mutant population of *Chlamydomonas* by transformation with a linearized plasmid conferring paromomycin or zeocin resistance, and we identified mutants with photosynthetic defects (*i*.*e*., acetate-requiring and/or light-sensitive and reactive oxygen species-sensitive mutants) [7,26]. However, we were only able to obtain FSTs for 17% of the mutants using PCR-based approaches. Here we employed low-coverage WGS of a subset of 660 mutants to identify the plasmid insertion sites and accompanying structural variants, and we found 1470 genes that are affected by the plasmid insertion in 509 mutants. We generated a list of 253 genes from 328 mutants that we refer to as higher-confidence causative genes, enabling the discovery of 183 potential photosynthesis genes: 169 genes of previously unknown function and 14 genes previously shown to have functions unrelated to photosynthesis. We experimentally validated two genes, *CrLPA3* and *CrPSBP4*, that are required for photoautotrophic growth in *Chlamydomonas*. In addition, our data provide insight into the spectrum of mutations that are induced by insertional mutagenesis in *Chlamydomonas*.

## Results

### Identification of insertion sites by mapping of discordant read pairs

**Fig 1:**
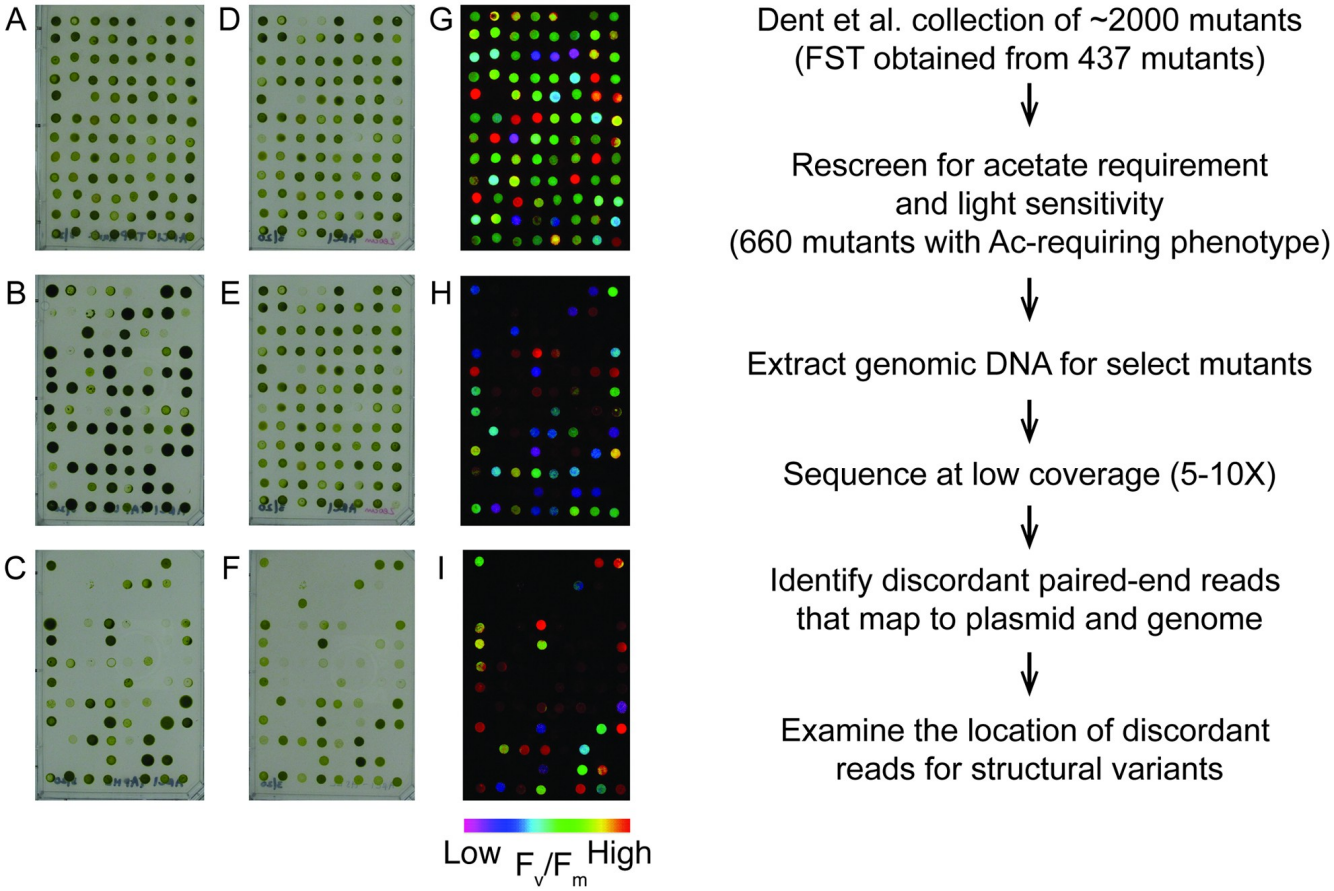
Growth and chlorophyll fluorescence screen pipeline. Mutants were scored for growth on (A) D+ac, (B) LL+ac, (C) HL+ac, (D) LL+ac+zeocin, (E) LL-min, (F) HL-min. F_v_/F_m_ values were measured on cells grown on (G) D+ac, (H) LL-min, (I) HL-min. The false color scale is indicated below the images (G-I). FST, flanking sequence tag. A representative plate spotted from a 96-well plate is shown. D, dark; LL, low light; HL, high light; +ac, added acetate; min, minimal media.

We re-screened our *Chlamydomonas* photosynthetic mutant collection [7,26] for growth on minimal and acetate-containing media under three light conditions (dark, D; low light of 60–80 μmol photons m^-2^ s^-1^, LL; and high light of 350–400 μmol photons m^-2^ s^-1^, HL) and for maximum photochemical efficiency of photosystem (PS) II (F_v_/F_m_) (S1 Table). An example of the phenotyping is shown in Fig 1. A total of 660 mutants, most of them with a growth phenotype and with resistance to either zeocin or paromomycin, indicative of the presence of the linearized plasmid sequence used for insertional mutagenesis, were chosen for WGS and herein will be referred to as the Acetate-Requiring Collection (ARC).

Genomic DNA was extracted from the 660 ARC mutants and submitted for low-coverage, paired-end WGS with a target depth of sequence coverage for each mutant between 5 and 10. The average sequencing depth across samples was 7.44. Paired-end reads that showed one end mapping to the plasmid used for mutagenesis and the other to a chromosome location were used to identify the plasmid insertion site(s) in each mutant. Plasmid insertion sites were not identified for 72 mutants, because few plasmid sequence reads were detected or the other end mapped to a low complexity region of the *Chlamydomonas* genome. 79 mutants had insertions that were not unique within the population (33 were duplicated, three were triplicated and one was quadruplicated) and were removed from further analysis. The remaining 509 mutant sequences were further analyzed for structural variants (insertions, deletions, and rearrangements) that occurred during insertional mutagenesis.

**Fig 2:**
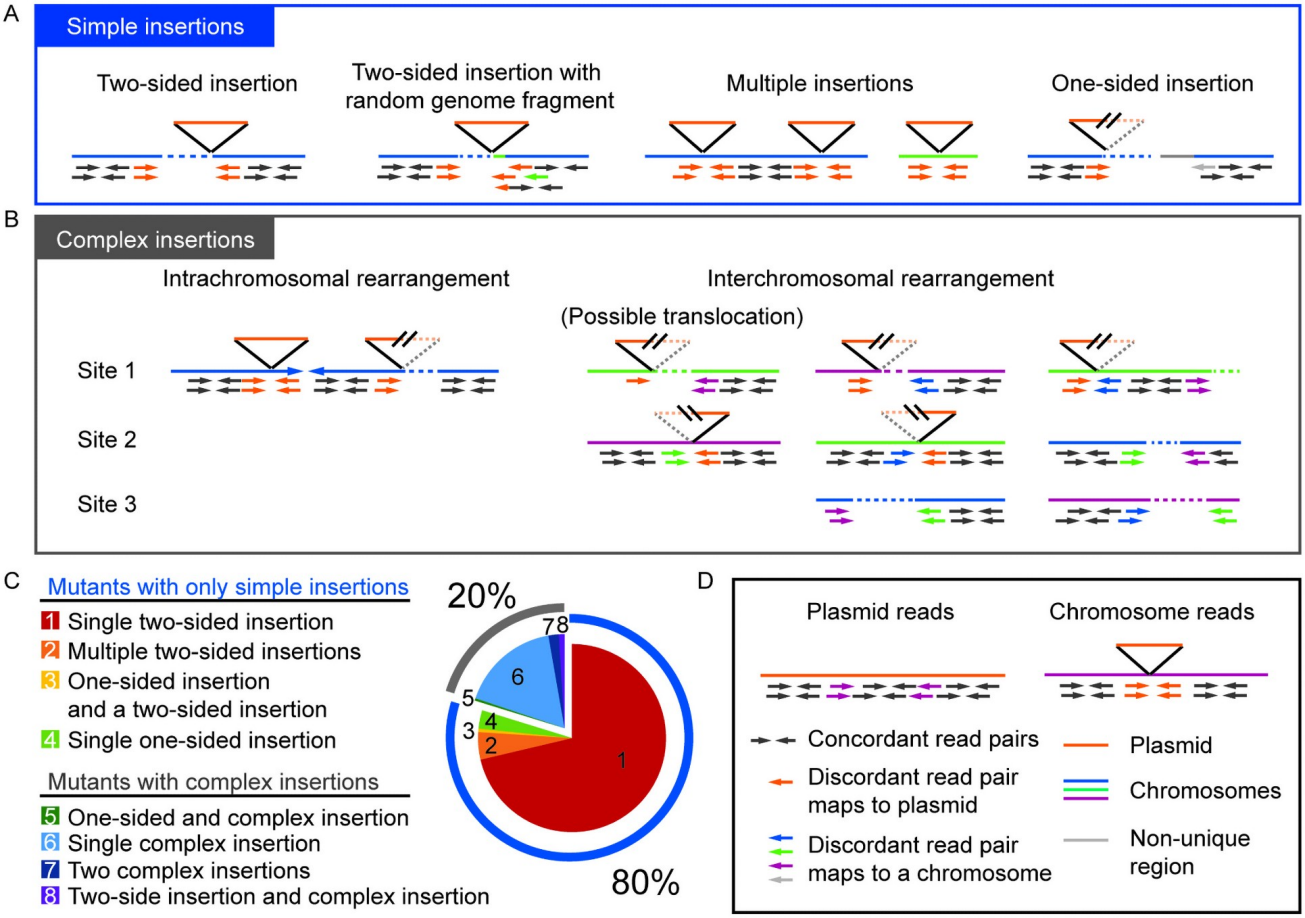
Examples of structural variations and the frequency of mutants with simple or complex insertions in ARC. Boxes contain schematic examples of mapped reads as seen in IGV. Black box, mapped reads (concordant and discordant) against plasmid and chromosome. Blue box, examples of “Simple insertions”; Gray box, examples of “Complex insertions”. Gray box shows examples of different complex insertions that are intra- or interchromosomal rearrangements. Second from left in gray box shows a possible translocation between two chromosomes. Pie chart shows frequency of “Simple mutants” containing only simple insertions and “Complex mutants” containing complex insertions.

Fig 2 illustrates the types of structural variants detected by analysis of the paired-end sequence data. Most sequence read pairs were concordant, i.e., they showed the expected orientation and distance with respect to each other when mapped to the *Chlamydomonas* genome (Fig 2, dark gray arrows). In contrast, discordant pairs showed the incorrect orientation or distances that were closer or further from each other than expected based on the genome fragmentation that was performed during sequencing library preparation (genomic DNA was sheared to approximately 600 bp) or on different chromosomes. In Fig 2, the discordant reads are shown as colored arrows, with each color representing a chromosome (or plasmid) to which the corresponding paired-end read was mapped. Each of these genomic sites where sequence read pairs were discordant is listed in S1 Table as a “Discordant site”. A total of 681 discordant sites were identified as being associated with plasmid insertions in the 509 mutants.

#### Defining insertions by whether they are simple or complex (i.e., involving rearrangements)

At most of the plasmid insertion sites, two sets of discordant read pairs were found, with their chromosomal reads oriented toward each other and their paired-end reads mapping to the plasmid sequence (Fig 2A). We refer to these 425 events as two-sided insertions, where both sides of the plasmid insertion were unambiguously mapped (S1 Table, column “Number of sides paired with plasmid at site”, 2). Another large group of discordant sites displayed only one set of discordant read pairs located on one side of the plasmid insertion (referred to as one-sided insertions Fig 2 and S1 Table, column “Number of sides paired with plasmid at site”, 1). The read-pairs on the other side of the plasmid insertion could not be mapped in 21 of these insertion sites because (i) it was at a repetitive region (14 mutants) and (ii) it had no discordant reads (7 mutants). These 21 one-sided insertions together with the 425 two-sided insertions making a total of 446 insertions were considered to be simple insertions (S1 Fig and S1 Table). In the rest of the one-sided insertions, the other side of the plasmid insertion paired with another chromosomal region indicating an occurrence of a more complex chromosomal rearrangement. An insertion that paired with another chromosomal location was considered a complex insertion. The frequencies of two-sided, one-sided, and complex insertions are shown in S1 Fig. Whether or not the discordant site paired with another site within the same mutant is listed in the column “Pairing with other discordant site(s) in the same mutant,” and such paired sites were considered to comprise a single lesion. The columns “Number of discordant sites for the mutant” and “Number of lesions for the mutant” specify these numbers for a single mutant.

#### Mutants that contain only simple insertions and those that contain a complex insertion

A total of 406 out of 509 mutants (80%) contained only simple insertions accounting for 435 out of the total 446 simple insertions (Fig 2C, Mutants with only simple insertions) (the remaining 11 simple insertions existed in mutants that also contained complex insertions). Among these 406 mutants, 24 mutants had multiple two-sided insertions accounting for 50 insertions, and three mutants had one two-sided insertion and one one-sided insertion (Fig 2C and S1 Fig). In 17 mutants, the multiple simple insertions occurred on the same chromosome, and six of these had tandem two-sided insertions that disrupted the same or neighboring genes. In 10 two-sided insertions (~1.8%), there appeared to be a short random fragment of another chromosome inserted together with the plasmid (Fig 2A, Two-sided insertion with random genome fragment). The original locus of these random fragments did not show a lack of mapped sequence reads but rather showed double the abundance of reads mapping to the small region, indicating that it was an extra copy of the same sequence at the insertion site, similar to what was observed in a previous study but at a lower frequency in ARC [8].

The other group of 103 mutants (20%) contained at least one complex insertion (Fig 2C “Mutants with complex insertions” and S1 Table, “Pairing with other discordant site(s) of the same mutant”). Some of these rearrangements occurred on a single chromosome, and others involved two or more chromosomes (Fig 2B). Among interchromosomal rearrangements, 13 of them involved two one-sided insertions that were paired to each other (Fig 2 gray box). These together may represent chromosomal translocation events resulting in two chimera chromosomes. In all of these possible translocation events, the plasmid sequence was present in one junction and not in the other. The proportion of complex insertion events was similar among the three plasmids used for transformation (pSP124S, pMS188, and pBC1). Validation of these complex structural variants would require *de novo* assembly of sequencing reads. Most mutants contained only two-sided or only complex insertions; 387 mutants (76%) had only two-sided insertion(s) (Fig 2C, red and orange slices), 92 had only complex insertion(s) (18%) (Fig 2C, light blue slice), and only a small proportion of mutants contained a mix of two-sided, one-sided, or complex insertions. Understanding whether or not a mutant contains only simple insertions is important in the search for causative mutations as discussed in following sections.

In summary, low-coverage WGS data for 509 ARC mutants identified 406 mutants that contained only simple insertions, whereas 103 mutants contained complex insertions that were associated with chromosomal rearrangements such as inversions and translocations.

### Analysis of deletions and duplications associated with insertional mutagenesis

**Fig 3:**
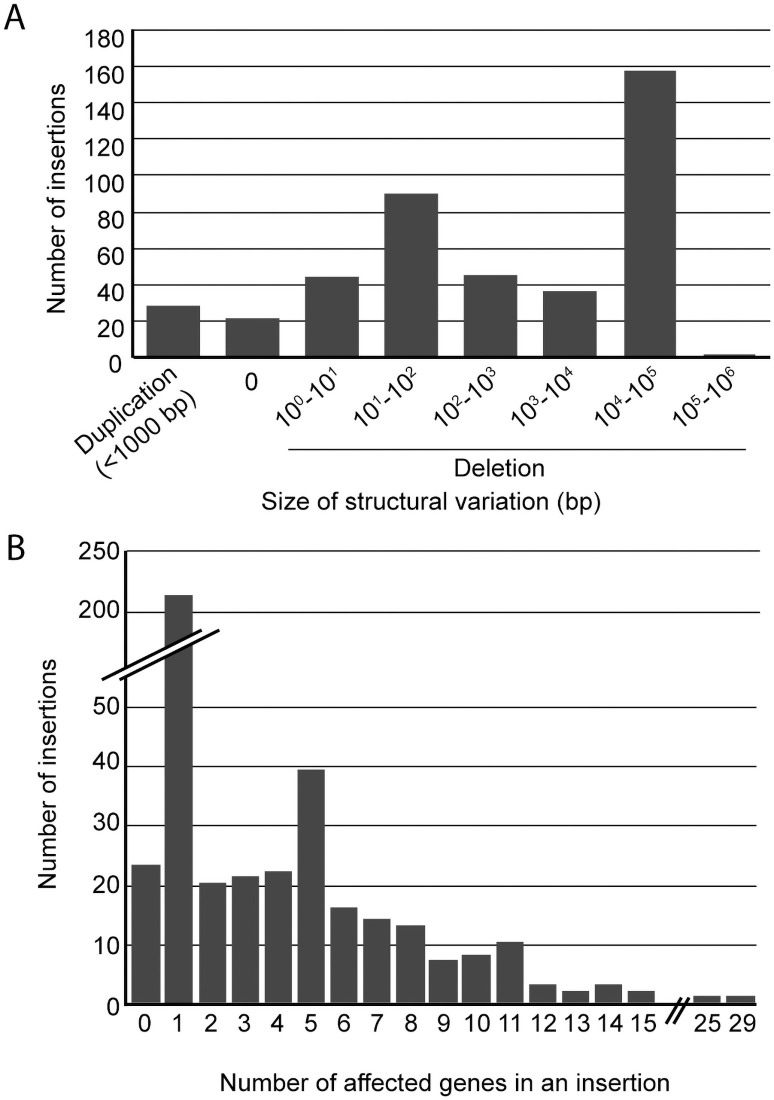
Structural variation accompanying insertions. (A) Duplication and deletion sizes and (B) number of mutants grouped by the number of genes affected by two-sided insertions. Only two-sided insertions were included in this analysis.

Insertional mutagenesis in *Chlamydomonas* has been previously associated with deletions and duplications at the site of plasmid insertion, especially when using glass bead for transformation (e.g. *cpld38*, *cpld49*, *npq4*, *rbd1*) [27–29]. Focusing on the 425 two-sided insertions, we found deletions associated with 374 insertions (88%). A wide range of deletion sizes was observed, with a bimodal distribution peaking at 11–100 bp and 10–100 kb when plotted at log_10_-scale, the largest deletion being 133 kb (Fig 3A). Duplications occurred less frequently (7%), in a total of 29 insertion events (Fig 3B), and all were less than 1000 bp. Perfect insertions lacking any duplications or deletions were found in only 22 events (5%). Despite the high frequency and relatively large size of many deletions, more than half (220 insertions) of the entire set of 425 two-sided insertions affected only a single gene (Fig 3C).

### Genetic linkage between acetate-requiring phenotype and antibiotic resistance

To determine if the phenotype of ARC mutants was likely caused by the plasmid insertion, we back-crossed 89 mutants to the wild type (WT) and analyzed the genetic linkage of the acetate-requiring phenotype and antibiotic (paromomycin) resistance in the respective progenies. The acetate-requiring phenotype was closely linked to the antibiotic resistance in 88% (77 out of 88 that produced viable zygospores) of mutants that were tested (S1 Table, column “Genetic Linkage”). In each cross, approximately 100 zygospores were collected and tested for recombination between the acetate-requiring phenotype and paromomycin resistance by selecting for progeny that were able to grow on minimal medium with paromomycin (S2 Fig). The lack of recombination and therefore growth indicates that the genetic distance between the mutation causing the acetate-requiring phenotype and paromomycin resistance is less than 0.5 cM, estimated to be 50 kb on average in the *Chlamydomonas* genome [15].

### Identification of secondary mutations using WGS data

In addition to the deletions associated with plasmid insertions in the ARC mutants, we searched for and found 68 other deletions using Pindel [30] (S2 Table). The size of the deletions ranged from 20 bp to 36 kb, with a majority of them (55 deletions, 77 genes) being less than 100 bp (S2 Table). The deletions were visually confirmed on alignments as direct gaps in reads and/or the lack of reads within the region, depending on the size. This was not expected to be an exhaustive search for such deletions. For example, low-coverage regions could be difficult to distinguish from a deletion. Nevertheless, some of the deletions affected clear candidate genes that could be responsible for the mutant phenotype. For example, the CAL014_01_19 mutant was found to contain a 21-bp deletion in Cre01.g013801, a GreenCut2 gene (conserved within genomes of land plants and green algae but absent from non-photosynthetic organisms [15,31]) annotated as a tocopherol cyclase (*VTE1*). The deletion occurred at the junction of intron 7 and exon 8, which could affect splicing and translation of a functional protein (S3 Fig). Because tocopherols are important for photoprotection in *Chlamydomonas* [32] disruption in the *VTE1* gene could explain this mutant’s high light-sensitive phenotype (S1 Table). In support of this hypothesis, a second mutant in the ARC, CAL033_02_19, had a 33-bp deletion in this locus. Interestingly, this mutant has a less severe phenotype (S1 Table), consistent with the plasmid insertion and deletion positioned in the 3’-UTR of the gene, which may have led to a partial loss of function (S3 Fig).

Among the 11 mutants whose acetate-requiring phenotype did not cosegregate with its paromomycin resistance, one (CAL036_02_12) had a strong acetate-requiring phenotype (S1 Table) and contained a secondary 36-kb deletion unassociated with the plasmid sequence and located 2 Mb away from the plasmid insertion on chromosome 7 (S2 Table). This resulted in a deletion of seven genes (Cre07.g346050, Cre07.g346100, Cre07.g346150, Cre07.g346200, Cre07.g346250, Cre07.g346300, and Cre07.g346317). One of these (Cre07.g346050) is *COPPER RESPONSE DEFECT 1* (*CRD1*), and *crd1* mutants have a conditional phenotype, lacking accumulation of PSI only under copper deficiency [33]. Another mutant (CAL029_03_36) has a one-sided insertion in *CRD1* and was only modestly affected in growth in HL (S1 Table), suggesting that the loss of CRD1 is not the sole cause of the severe growth phenotype of CAL036_02_12. Another one of the deleted genes is annotated as phytol kinase (Cre07.g346300). Chlorophyll degradation and phytol remobilization through phytol kinase (*VTE5*) and phytol phosphate kinase (*VTE6*) are important for α-tocopherol biosynthesis and their disruption results in high light sensitivity in tomato [34] and *Arabidopsis* [35]. The light sensitivity observed in CAL036_02_12 is similar to that of tomato plants silenced for *VTE5* [34] and strongly suggests that Cre07.g346300 is the causative gene for the mutant phenotype. The remaining 10 mutants whose acetate-requiring phenotype is unlinked to the plasmid insertion would be candidates for higher-coverage WGS to search for causative mutations.

### Genes with multiple mutant alleles in the ARC

In total, 1404 genes were directly affected by the 554 plasmid insertions in 509 mutants. There are many more affected genes compared to the number of mutants from which they originate due to disruption of multiple genes by large deletions. Additionally, among the 77 genes affected by deletions that were unassociated with plasmid insertions (S2 Table), 11 overlapped with the 1404 genes, bringing the total number of genes disrupted in our library to 1470. S3 Table lists all of the 1470 genes and their available annotations.

**Fig 4:**
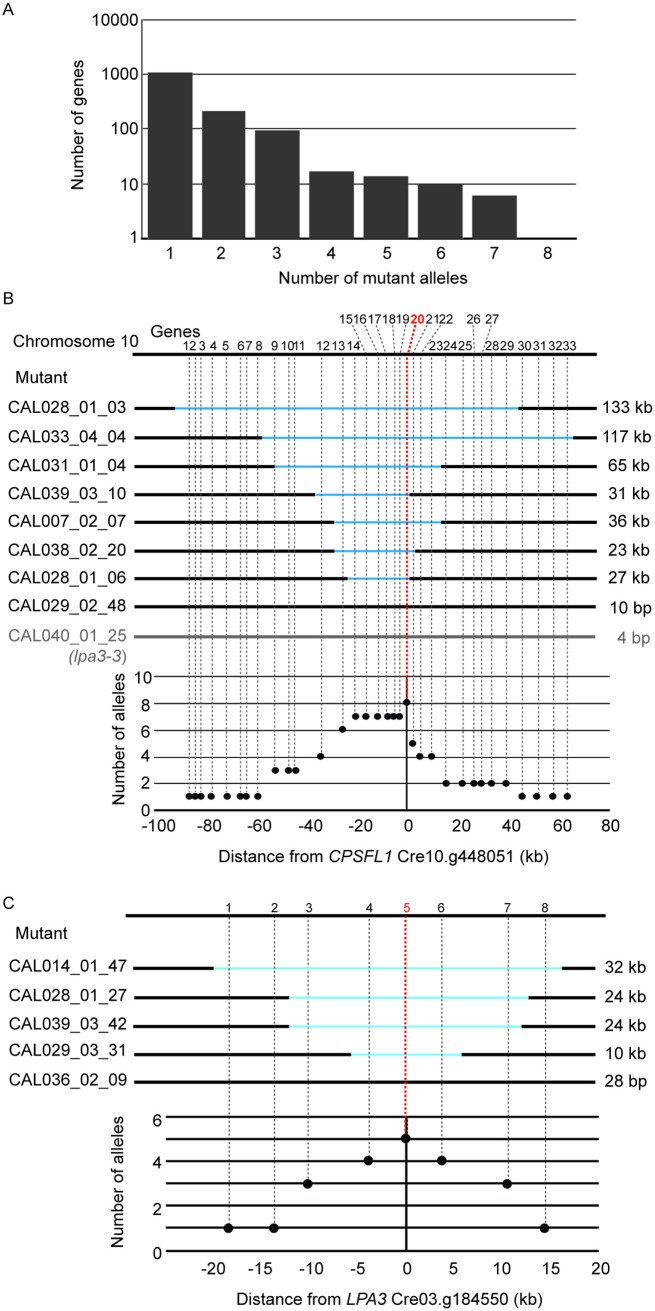
Genes represented by multiple mutant alleles are more likely to be causative genes. (A) Number of genes affected by plasmid-associated insertions in ARC grouped by the number of mutant alleles that represent the gene. Schematic of mutant alleles disrupted in (B) *cpsfl1* mutants and (C) *lpa3* mutants and the allele frequencies of surrounding genes. Note that not all genes with multiple mutant alleles are causative; some genes belong to this group because of their physical proximity to the true causative genes. CAL040_01_25 (*lpa3-3*, Fig 5) is indicated in gray because this mutant is not included in S1 Table or the analysis for panel A but represents the ninth mutant allele of *cpsfl1*.

To begin identifying causative mutations, we searched for genes that were affected in multiple ARC mutants. Fig 4A shows the number of alleles of each gene affected by plasmid-associated insertions (S1 Table). Interruption/deletion of 1053 genes only occurred once, while 212 genes have two alleles and 94 genes have three alleles. Some genes appeared on the list of affected genes more than three times (Fig 4A). However, because disruption of multiple genes occurred in approximately half of the ARC mutants, many of these genes represented by multiple alleles are likely not causative for the mutant phenotype. Some of the genes appear more frequently on the list simply because of their proximity to the causative gene. Fig 4B shows an example of such an occurrence for *CPSFL1* (Cre10.g448051). Seven ARC mutants had deletions ranging from 22 to 130 kb in a region on chromosome 10 (CAL028_01_03, CAL033_04_04, CAL031_01_04, CAL039_03_10, CAL007_02_07, CAL038_02_20, and CAL028_01_06) (S1 Table). 33 genes were affected by the deletions in these mutants, including seven genes affected in all seven mutants, which makes it difficult to narrow down to a single causative gene. One additional mutant (CAL29_02_48) had a complex insertion event involving four different chromosomes, but strikingly it shared a single affected gene (*CPSFL1*, containing a 10-bp deletion) with the other seven mutants. All eight mutants exhibited a strict acetate-requirement and severe light-sensitivity phenotype (S1 Table), and in-depth characterization of the CAL028_01_06 mutant showed that *CPSFL1* is involved in carotenoid accumulation and is essential for photoautotrophic growth in *Chlamydomonas* and *Arabidopsis* [36,37].

The *CrLPA3* gene (Cre03.g184550, hereon *LPA3*) is another example of a gene that was affected in multiple mutants (Fig 4C). The CAL014_01_47, CAL028_01_27, CAL039_03_42, CAL029_03_31, and CAL036_02_09 mutants had overlapping deletions ranging from 28 bp to 32 kb in the same region on chromosome 3, and all five mutants exhibited a strict acetate-requiring phenotype in HL (S1 Table). By comparing the disruption frequencies, we identified *LPA3* as the only gene that was affected in all five mutants.

### *LPA3* and *PSBP4* are essential for photoautotrophic growth and accumulation of the photosystems

**Fig 5:**
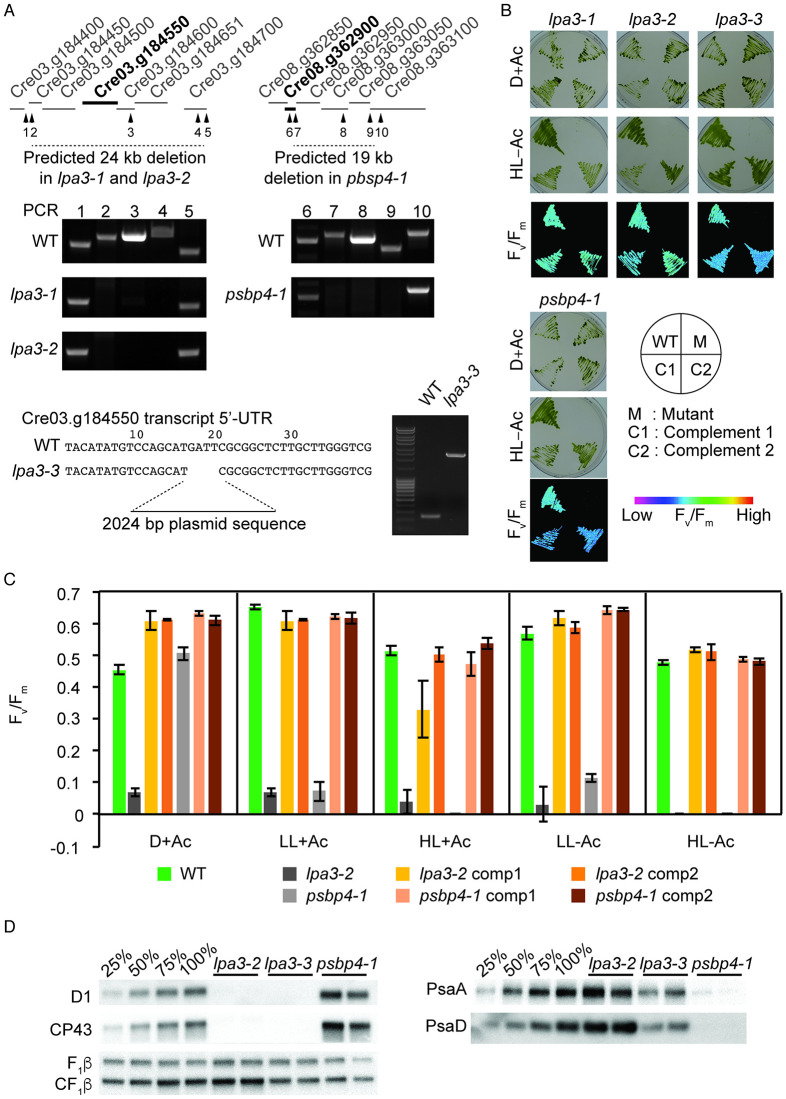
Identification of *CrLPA3* and *CrPSBP4* required for photoautotrophic growth. (A) Schematic of loci and deletions indicated from whole-genome sequence data in mutants *lpa3-1* (CAL028_01_27), *lpa3-2* (CAL039_03_42), and *lpa3-3* (CAL040_01_25) that share a disruption in Cre03.g184550, gene encoding a predicted ortholog of Arabidopsis LOW PHOTOSYSTEM II ACCUMULATION 3 (LPA3) and mutant *psbp4-1* (CAL032_04_48) that had a deletion encompassing Cre08.g362900, a gene encoding a protein predicted as PSBP4. Numbered arrowheads indicate the PCR probes used in testing for deletions shown in the agarose gel photos. WT and *lpa3-3* sequences indicate the plasmid insertion site and associated 4 bp-deletion. (B) Growth and chlorophyll fluorescence phenotype of WT, mutants and their complemented lines. Images are representative of an experiment repeated twice. Cells were grown with acetate in the dark or without acetate under 400 μmol photons s^-1^ m^-2^ and imaged for growth and F_v_/F_m_ measurements (HL-Ac). F_v_/F_m_ value are represented by false colors as shown in the reference bar. (C) F_v_/F_m_ values of each genotype under different growth conditions. Values indicate averages of three biological replicates; error bars represent standard deviations. comp, complemented line. (D) PSII and PSI subunit accumulation shown by immunoblotting against subunits of PSII (D1 and CP43) and PSI (PsaA and PsaD). Mitochondrial ATP synthase (F_1_β) was probed as loading control with an antibody that also detects the F_1_β subunit of the chloroplast ATP synthase (CF_1_β). Each genotype was analyzed in biological duplicates (one per lane). Dilutions of the WT samples are shown in the four left lanes.

We proceeded to validate the WGS data and identify two genes as necessary for photoautotrophic growth in *Chlamydomonas*. In one case (*LPA3*), multiple alleles were present in the ARC, whereas only a single allele of the other gene *CrPSBP4* (hereon *PSBP4*) was present. Three *lpa3* mutants (CAL028_01_27, CAL039_03_42, and CAL040_01_25) were selected for further analysis (and renamed as *lpa3-1*, *lpa3-2*, and *lpa3-3*, respectively). The WGS data indicated that the *lpa3-1* and *lpa3-2* mutants had very similar deletions of 24 kb that affected the same five genes (S1 Table). The deletion was confirmed by amplifying genomic regions across the predicted deletion by PCR in both mutants (Fig 5A), although it was not possible to amplify the plasmid sequence at the site of the deletion. The *lpa3-3* mutant was predicted from WGS to have a 4-bp deletion and plasmid insertion in the 5’-UTR of Cre03.g184550, which was confirmed by sequencing a PCR fragment of the region from the mutant (Fig 5A), but it was not included in S1 Table, because it was one of the 79 mutants with a non-unique insertion site (see above in section “Identification of insertion sites by mapping of discordant read pairs”). All three mutants had an acetate-requiring phenotype (Fig 5B). The gene Cre03.g184550 encodes a GreenCut2 protein (CPLD28) [31], and is annotated as an ortholog of *Arabidopsis* LOW PSII ACCUMULATION 3 (LPA3). *Arabidopsis* LPA3 has been reported to be involved in the assembly of photosystem II [38], although the publication on the function of this protein was later retracted [39]. Complementation with a genomic DNA clone of Cre03.g184550 (*LPA3*) including 1.2 kb upstream of the transcription start site rescued all three mutants, demonstrating that the disruption of this gene was responsible for the acetate-requiring phenotype of these mutants. Mutants lacking LPA3 exhibited very low F_v_/F_m_ values even in the dark (Fig 5C). This suggests that *Chlamydomonas* LPA3 is required for the assembly of PSII even in the absence of light, resulting in a much more severe phenotype than *lpa3* single mutants in *Arabidopsis* that accumulate PSII-LHCII supercomplexes at a slower rate than WT plants [38]. PSII subunits did not accumulate in *lpa3* mutants grown in TAP under very low light (0–2 μmol photons m^-2^ s^-1^) (Fig 5D). A concomitant overaccumulation of PSI subunits and ATP synthase was observed in *lpa3-2* but not in *lpa3-3*, in which the abundance of PSI subunits was reduced as compared to WT (Fig 5D). This may be due to allele-specific differences; *lpa3-2* is a complete knock-out as compared to *lpa3-3*, which has an insertion in the 5’-UTR. The low F_v_/F_m_ phenotype of the mutants was rescued in the complemented lines in all light conditions (Fig 5B and 5C).

The mutant CAL032_04_48 (renamed as *psbp4-1*) required acetate for growth and exhibited light sensitivity even in the presence of acetate, and its F_v_/F_m_ was reduced compared to that of the WT when grown in the light (S1 Table and Fig 5C). Its WGS indicated two tandem simple insertions disrupting five genes. Among them, Cre08.g362900, annotated as encoding a thylakoid luminal PsbP-like protein (PSBP4), presented itself as a clear candidate to be the gene responsible for the phenotypes. The PSBP4 ortholog of *Arabidopsis* has been shown to be involved in the assembly of PSI [40,41]. The deletion in *psbp4-1* was confirmed by PCR (Fig 5A). Immunoblotting showed that the mutant failed to accumulate PSI subunits to wild-type levels but overaccumulated PSII subunits (Fig 5D). The mutant growth and F_v_/F_m_ phenotypes were rescued by transforming with genomic DNA including Cre08.g362900 and upstream region, demonstrating that disruption of *PSBP4* was the cause of the acetate-requiring and light-sensitive phenotypes of this mutant (Fig 5B and 5C).

### Curation of higher-confidence photosynthesis candidate genes

To identify candidate genes that are likely to be responsible for the ARC mutant phenotypes, we focused on the 406 mutants with only simple insertions (Fig 2C). We reasoned that when considering the causative gene of a mutant phenotype, a mutant with only simple insertion events is more likely to have a causative gene within its disrupted gene list than a mutant with a complex insertion event that is accompanied by large-scale chromosomal rearrangements, which could cause unpredictable changes in expression of neighboring genes due to alterations in promoters, enhancers, and chromatin environment. For each of the 406 mutants with simple insertions, we applied a series of criteria to generate a list of genes that are the strongest confidence candidates for being genes that are responsible for the ARC mutant phenotype. If a mutant contained a single, simple insertion that disrupts a single gene then that gene was immediately considered to be a higher-confidence candidate, based on the 88% genetic linkage observed between paromomycin resistance and acetate requirement (S2 Fig). If a mutant contained a simple insertion with multiple genes disrupted by an associated deletion, then we manually analyzed the genes and selected the best candidate, considering whether it was a GreenCut2 gene, whether it was co-expressed with photosynthesis genes [42], and whether it encoded a protein with annotation or domains indicating a possible function in photosynthesis (e.g. redox, chlorophyll *a/b*-binding, Fe-S cluster). 78 GreenCut2 genes that were disrupted in 509 ARC mutants (Table 1) and were considered strong candidates unless there was an even stronger candidate based on functional annotation. As was shown for *cpsfl1* (Fig 4B) and *lpa3* (Fig 4C), mutants with overlapping disrupted genes were also compared to find the strongest candidate (gene with highest disruption frequency). Neighboring genes that were co-disrupted with the strongest candidates were deemed non-candidates in all the mutants. As a final criterion, we searched candidate genes derived from analysis of other existing photosynthesis mutant libraries and identified overlaps with *Chlamydomonas* genes whose disruption affected photoautotrophic growth (Chlamydomonas Library Project, CLiP) [9], orthologous genes from the maize Photosynthetic Mutant Library (PML, http://pml.uoregon.edu/pml_table.php) [43], and orthologous genes identified from Dynamic Environmental Photosynthetic Imaging (DEPI) of *Arabidopsis* mutants [44]. Additionally, we surveyed the genes affected by small deletions unassociated with the plasmid insertion (S2 Table) with the same set of criteria. Two mutants were identified as additional alleles of candidate causative genes as well as two new candidates and their corresponding mutants.

**Table 1:** GreenCut2 proteins within genes affected in ARC.

Gene ID	Gene name	Description	Comments
Cre01.g000850	CPLD38	Required for cyt *b*_*6*_*f* accumulation	
Cre01.g009650	BUG25	Basal body protein and putative AP2 domain transcription factor	
Cre01.g013801	VTE1	Tocopherol cyclase	
Cre01.g016500		Dihydrolipoamide dehydrogenase	Not in Table 2
Cre01.g016514	DLD2	Dihydrolipoamide dehydrogenase	
Cre01.g027150		DEAD/DEAH-box helicase	
Cre01.g033763		D-Amino acid aminotransferase-like PLP-dependent enzymes superfamily	
Cre01.g033832		DEAD-box ATP-dependent RNA helicase 39	
Cre01.g043350	CAO1	Chlorophyllide *a* oxygenase	
Cre01.g049000		Pterin dehydratase	
Cre01.g049600	CGLD22	Expressed protein similar to ATP synthase I	
Cre02.g084350	CGLD1	Predicted protein	
Cre02.g084500		Zinc finger MYND domain containing protein 10	Not in Table 2
Cre02.g084550	NAT10	Acyl-CoA N-acyltransferase-like protein	Not in Table 2
Cre02.g086550	CGL122	23S rRNA (adenine2503-C2)-methyltransferase	
Cre02.g105450	CGL141	F7O18.3 PROTEIN	Not in Table 2
Cre02.g114750	CDPK5	MAP kinase activated protein kinase 5	Not in Table 2
Cre02.g120100	RBCS1	RubisCO small subunit 1, chloroplast precursor	
Cre02.g120150	RBCS2	RubisCO small subunit 2	
Cre03.g158900	DLA2	Dihydrolipoamide acetyltransferase	
Cre03.g160300	RAM1	Stress associated endoplasmic reticulum protein SERP1/RAMP4	Not in Table 2
Cre03.g173350	ANK22	Predicted protein with ankyrin repeats	Not in Table 2
Cre03.g182551	PCY1	Pre-apoplastocyanin	
Cre03.g182600	CPL1	Histone deacetylation protein Rxt3	Not in Table 2
Cre03.g184550	CPLD28	LPA3, Predicted protein	
Cre03.g185200		Metallophosphoesterase/metallo-dependent phosphatase	
Cre05.g246800	GUN4	Tetrapyrrole-binding protein	
Cre05.g243800	CPLD45	PSB27	
Cre05.g242400	PGR5	Proton Gradient Regulation 5, chloroplastic	
Cre05.g242000	CHLD	Magnesium chelatase subunit D	
Cre05.g238332	PSAD	Photosystem I reaction center subunit II	
Cre06.g278212	CGL46	Predicted protein	Not in Table 2
Cre06.g280650	CGL59	Predicted protein	
Cre07.g315150	RBD1	Rubredoxin	
Cre07.g318200	CGLD34	ET and MYND domain-containing protein DDB	
Cre08.g362900	PSBP4	Lumenal PsbP-like protein	
Cre08.g372000	CGLD11	Predicted protein	
Cre08.g382300	CCB4	CGLD23 protein	
Cre09.g387000	CGL34	Predicted protein	Not in Table 2
Cre09.g394325	ELI3	Early light-inducible protein	
Cre09.g411200	TEF5	Rieske [2Fe-2S] domain containing protein	
Cre10.g420350	PSAE	Photosystem I 8.1 kDa reaction center subunit IV	
Cre10.g435850	CPLD24	Predicted protein	Not in Table 2
Cre10.g440450	PSB28	Photosystem II subunit 28	
Cre10.g445100	CGL50	Predicted protein	
Cre10.g466500	CPL12	Glyoxylase family protein (yaeR)	
Cre11.g467689	PETC	Rieske iron-sulfur subunit of the cytochrome *b*_*6*_*f* complex, chloroplast precursor	
Cre11.g467754		Solute carrier protein, UAA transporter family	Not in Table 2
Cre11.g467700	UPD1	Uroporphyrinogen-III decarboxylase	
Cre11.g468750	CPLD48	Predicted protein	Not in Table 2
Cre11.g469450	CGL124	Adhesion regulating molecule 110kDa cell membrane glycoprotein	
Cre12.g494000	CGL82	Predicted protein	
Cre12.g510050	CTH1	Copper target 1 protein	Not in Table 2
Cre12.g509050	PSBP3	OEE2-like protein of thylakoid lumen	
Cre12.g517700		Short-chain dehydrogenase/reductase, probably chlorophyll *b* reductase	
Cre12.g524300	CGL71	Predicted protein	
Cre12.g524350	HUS1	DNA damage checkpoint protein	Not in Table 2
Cre12.g554800	PRK1	Phosphoribulokinase	
Cre13.g562475		ER lumen protein retaining receptor family protein-related	Not in Table 2
Cre13.g563150	CGLD8	Predicted protein	
Cre13.g575000	CCS1	Protein required for cytochrome *c* synthesis/biogenesis	
Cre13.g577850		Peptidyl-prolyl *cis-trans* isomerase, FKBP-type	Not in Table 2
Cre13.g578650		Similar to complex I intermediate-associated protein 30	Not in Table 2
Cre13.g579550	CGL27	Predicted protein	Not in Table 2
Cre14.g618050	PLP3	Plastid lipid associated protein	Not in Table 2
Cre14.g624201		Thioredoxin-like protein CDSP32, chloroplastic	Not in Table 2
Cre16.g660000	CPLD63	GDT1-like protein 2, chloroplastic	Not in Table 2
Cre16.g665250	APE1	Thylakoid associated protein, Acclimation of Photosynthesis to Environment1	
Cre16.g666050	CPLD49	Saccharopine dehydrogenase	
Cre16.g687450	CPLD54	K^+^ Efflux Antiporter 3, chloroplastic (KEA3)	Not in Table 2
Cre16.g675100	CPLD53	Zinc finger protein Constans-related	
Cre16.g674950	POD2	Prolycopene isomerase / CRTISO	Not in Table 2
Cre17.g702150	HCF164	Thioredoxin-like protein HCF164, chloroplastic	
Cre17.g702500	TAB2	PsaB RNA binding protein	
Cre17.g710800	NFU3	Iron-sulfur cluster assembly protein	
Cre17.g717350	TRI1	tRNA dimethylallyltransferase / tRNA prenyltransferase	Not in Table 2
Cre17.g717400	TRIT1	tRNA dimethylallyltransferase (miaA, TRIT1)	
Cre17.g731100	CPL14	DUF2358	

We were able to identify 253 higher-confidence candidate genes which are shown in Table 2 (and with their corresponding mutants in S4 Table with additional details and references). This list includes genes known to be important for photosynthesis, photoprotection, and peripheral functions either in *Chlamydomonas* or other photosynthetic organisms (S4 Table, Column “Function reported in Cr and other photosynthetic organisms”). 103 gene products were predicted to be targeted to the chloroplast by protein targeting software Predalgo (https://giavap-genomes.ibpc.fr/cgi-bin/predalgodb.perl?page=main) [45], and among those, 78 were also predicted to be targeted to plastids by ChloroP (http://www.cbs.dtu.dk/services/ChloroP/) [46] (Table 2 and S4 Table). Proteins encoded by 31 genes were found in the chloroplast proteome of *Chlamydomonas* [47], 27 of which were also *in silico*-predicted chloroplast proteins. 53 GreenCut2 genes are within this higher-confidence list, leaving 25 GreenCut2 genes that were not chosen because there was a stronger candidate gene (see column “Comments” in Table 1). Among the 253 candidates, the photosynthetic functions of 70 genes have been previously described in *Chlamydomonas*, land plants, or cyanobacteria. 14 genes have been described as having functions other than photosynthesis, such as plant meristem development, sulfur response, or carbon metabolism (S4 Table, Known genes whose functions are not Photosynthesis). This leaves 183 genes whose functions remain to be studied in context of photosynthesis, 38 of which have no annotation (S4 Table).

**Table 2:** Higher-confidence photosynthesis candidate genes. Higher confidence was determined by manual curation of the genes disrupted in a mutant following multiple criteria: (i) single gene disruption in a simple mutant, (ii) highest frequency disruption among multiple mutant alleles, (iii) GreenCut2 membership, (iv) protein domains associated with photosynthetic functions.

Cre ID	Gene name	Description	Subcellular localization^1^	GreenCut2^2^	Other mutant libraries^3^	Multiple candidates^4^
Cre01.g000850	CPLD38	DUF3007	C	G		
Cre01.g009650	BUG25	Basal body protein and putative AP2 domain transcription factor	O	G		
Cre01.g013801	VTE1	Tocopherol cyclase	C, CP	G		
Cre01.g016514	DLD2	Dihydrolipoyl dehydrogenase/Lipoyl dehydrogenase	C	G		
Cre01.g016570		Mitogen-activated protein kinase kinase kinase 19	O			
Cre01.g018600	BAP31	B-cell receptor-associated protein 31-like	C			
Cre01.g019700	PAP7	Non-canonical poly(A) polymerase	O			
Cre01.g027150	CPLD40, HEL5	DEAD/DEAH-box helicase	C	G	Cr	
Cre01.g030700	PTK14	Protein tyrosine kinase	O			
Cre01.g033450		Sphingomyelin phosphodiesterase 2	O			
Cre01.g033763		D-Aminoacid aminotransferase-like PLP-dependent enzymes superfamily protein	C	G		
Cre01.g033832		DEAD-box ATP-dependent RNA helicase 39	C	G		
Cre01.g034600		WD-40 domain	C			
Cre01.g043350	CAO1	Chlorophyllide *a* oxygenase	C	G		
Cre01.g044850		Sacsin (SACS)	O			
Cre01.g049000	CGL31, PTD1	Pterin dehydratase	C	G	Zm	
Cre01.g049600	CGLD22	Expressed protein similar to ATP synthase I	C	G		
Cre01.g050500	PPR1	Pentatricopeptide repeat protein	C		Cr	
Cre01.g053900	NGLY1, PNG1	Peptide-N4-(N-acetyl-beta-glucosaminyl)asparagine amidase	O			
Cre02.g074900		Extended synaptotagmin-related	C		Cr	
Cre02.g076600		Peptidyl-tRNA hydrolase, PTH1 family	C		Zm	
Cre02.g080700	BIP1	Endoplasmic reticulum associated HSP70 protein	O			
Cre02.g084250	PPP7	Protein phosphatase 1K, mitochondrial	O			M
Cre02.g084350	CGLD1	Predicted protein (GDT1 like protein 1, chloroplastic)	O	G		M
Cre02.g086550	CGL122	23S rRNA (adenine2503-C2)-methyltransferase (rlmN)	C	G		
Cre02.g087900		Mitogen-activated protein kinase kinase kinase/MLTK	C			
Cre02.g088650		Phosphatidylinositol N-acetylglucosaminyltransferase/glucosaminyltransferase	O			
Cre02.g099601		Androgen induced inhibitor of proliferation AS3/PDS5-related	O			
Cre02.g099850	PDC2	Pyruvate dehydrogenase, E1 component, alpha subunit	C, CP			
Cre02.g100300		Phosphatidylinositol 3-kinase-related protein kinase	O			
Cre02.g105650	LPA2		C		Cr	
Cre02.g106250	LAL2	La-like RNA-binding protein	O			
Cre02.g110500			O			
Cre02.g120100	RBCS1	Ribulose-1,5-bisphosphate carboxylase/oxygenase small subunit 1, chloroplast precursor	C	G		M
Cre02.g120150	RBCS2	Ribulose-1,5-bisphosphate carboxylase/oxygenase small subunit 2	C	G		M
Cre02.g120250	CDPK7, STT7	Calcium/calmodulin-dependent protein kinase	C			M
Cre02.g142146		Divinyl chlorophyllide *a* 8-vinyl-reductase/[4-vinyl]chlorophyllide *a* reductase	C		Zm	
Cre02.g142750			O			
Cre02.g143400		3’,5’-cyclic-nucleotide phosphodiesterase	O			
Cre03.g145387	FAP239	Flagellar associated protein	O			
Cre03.g145987			O			
Cre03.g149450		Ion channel pollux-related	C			
Cre03.g154550	PCR1	Pyrroline-5-carboxylate reductase	C			
Cre03.g155250			C			
Cre03.g156150		ATP-dependent RNA helicase DDX10/DBP4	O			
Cre03.g158900	DLA2	Dihydrolipoamide acetyltransferase	C, CP	G		
Cre03.g159851		I-kappa-b-like protein IKBL	C			
Cre03.g160250			O			M
Cre03.g160400	SAC1	Sulfur acclimation 1 protein, sodium/sulfate co-transporter	O			M
Cre03.g164900		Serine/Threonine protein kinase OSR1	O			
Cre03.g172500	PTO2/PTOX2	Plastid terminal oxidase	C			
Cre03.g173600		Ubiquitin and ubiquitin-like proteins	O			
Cre03.g175700		CobW-related	O			
Cre03.g179650		BTB/POZ domain (BTB)	O			
Cre03.g182550	PNO3	Ferredoxin-NAD(+) reductase	O			
Cre03.g182551	PCY1	Pre-apoplastocyanin	C, CP	G	Cr	
Cre03.g182900		PNAS-related	O			
Cre03.g184550	CPLD28, LPA3	Predicted protein	O	G		M
Cre03.g185200	CPL3, MPA6	Metallophosphoesterase/metallo-dependent phosphatase	C	G	Cr	M
Cre03.g185550	SBP1	Sedoheptulose-1,7-bisphosphatase	C, CP		Cr	
Cre03.g194200	PDH2	Pyruvate dehydrogenase E1 beta subunit	C, CP			M
Cre03.g197450		Winged helix DNA-binding domain-containing protein	O			
Cre03.g199250	CYG51	Adenylate/guanylate cyclase	O			
Cre03.g206369		Tyrosine kinase specific for activated (GTP-bound)//Serine/Threonine protein kinase	C		Cr	
Cre03.g207153			C			
Cre03.g207400		von Willebrand factor type A domain	O			
Cre03.g209505		Serine/Threonine-protein kinase SRK2	O			
Cre03.g210961		Phosphatidylinositol transfer protein PDR16-related	O			
Cre03.g211633		Similar to Flagellar Associated Protein FAP165	C			
Cre03.g213201			C			
Cre04.g212401		Baculoviral IAP repeat-containing protein 6 (apollon) (BIRC6, BRUCE)	O			
Cre05.g232150	GDH2	Glutamate dehydrogenase	O			
Cre05.g232200	NDA3	Mitochondrial NADH dehydrogenase	C			
Cre05.g238322		Tryptophan-tRNA ligase/Tryptophanyl-tRNA synthetase	C			
Cre05.g238332	PSAD	Photosystem I reaction center subunit II, 20 kDa	C	G	Cr	
Cre05.g238500		23S rRNA (adenine2503-C2)-methyltransferase	C			
Cre05.g241900			C			
Cre05.g242000	CHLD	Magnesium chelatase subunit D	C, CP	G	Cr	
Cre05.g242400	PGR5		C	G		
Cre05.g243800	CPLD45	Predicted protein	C, CP	G	Cr	
Cre05.g246800	GUN4	Tetrapyrrole-binding protein	C	G	Zm	
Cre06.g259100			C		Cr	
Cre06.g262650	OPR22, TAA1	RAP domain (RAP)	C			
Cre06.g264100			O			
Cre06.g268750	MME1	Malate dehydrogenase, decarboxylating	O			
Cre06.g271200		NADH oxidase (H_2_O_2_-forming)	C			
Cre06.g278094	ELG14	Exostosin-like glycosyltransferase	O			
Cre06.g280050	XRN1	Single-stranded RNA 5’->3’ exonuclease	O			M
Cre06.g280150	PSBP9	PsbP-like protein	C			M
Cre06.g280650	CGL59	Predicted protein	C	G	Cr, Zm	
Cre06.g281250	CFA1	Cyclopropane fatty acid synthase	O			
Cre06.g281800		Domain of unknown function (DUF1995)	C		Cr	
Cre06.g284100	RHP1	Rh protein, CO_2_-responsive	C			M
Cre06.g284150	RHP2	Rh protein	C			M
Cre06.g289600			O			
Cre06.g300250	TTL10	Tubulin polyglutamylase TTLL2	O			
Cre06.g302305			O			
Cre06.g308100		Enoyl-CoA hydratase 2/ECH2	O			
Cre07.g315150	RBD1	Rubredoxin	C, CP	G		
Cre07.g318200	CGLD34	SET and MYND domain containing protein DDB	O	G		
Cre07.g336150			O			
Cre07.g342920		Xaa-Pro dipeptidase/X-Pro dipeptidase	O			
Cre07.g344950	LHCA9	Light-harvesting protein of photosystem I	C			
Cre07.g348550	TGL13	Protein T08B1.4, Isoform B-related (lipase related)	O			
Cre07.g349800			C			
Cre07.g355750		F-box and WD40 domain protein	O			
Cre07.g356350	DXS1	1-Deoxy-D-xylulose 5-phosphate synthase, chloroplast precursor	C, CP			
Cre07.g356450		Leucine-rich repeat-containing protein	O			
Cre08.g358250	MCA1	PPR repeat/Maturation/stability factor for petA mRNA	C		Zm	
Cre08.g358350	TDA1, OPR34	FAST Leu-rich domain-containing	C		Cr	
Cre08.g361250		Protein *O-*GlcNAc transferase/OGTase (DUF563)	C			
Cre08.g362900	PSBP4	Lumenal PsbP-like protein	C	G	Zm	
Cre08.g365200			O			
Cre08.g365550			O			
Cre08.g370550		D-2-Hydroxyglutarate dehydrogenase	O			
Cre08.g372000	CGLD11	Predicted protein	C, CP	G		
Cre08.g375000		Actin-fragmin kinase, catalytic	O			
Cre08.g382300	CCB4	CGLD23 protein, required for Cyt *b*_*6*_ assembly	C	G	Zm	
Cre08.g382515		WD repeat-containing protein 26	O			
Cre08.g385300		ET and MYND domain-containing protein DDB	O			
Cre09.g388356	TBC2	Translation factor for chloroplast psbC mRNA/Translation factor for chloroplast psbC mRNA	C		Cr	
Cre09.g390060			C		Cr	
Cre09.g391356		Mitogen-activated protein kinase kinase kinase/MLTK	O			
Cre09.g392729		Methionyl-tRNA formyltransferase/transformylase	C			
Cre09.g393136		Clathrin assembly protein	O			
Cre09.g394150	RAA1	FAST kinase-like protein, subdomain 1	C		Cr	
Cre09.g394325	ELI3	Early light-inducible protein	C	G		
Cre09.g397956	FAP201	Flagellar associated protein (Exotosin family)	O			
Cre09.g398919			C			
Cre09.g410000		DC12-Related	O			
Cre09.g411200		Rieske domain-containing protein	C, CP	G	At	
Cre10.g417750		Neuropathy target esterase/Swiss cheese *D*. *melanogaster*	C			
Cre10.g419250			O			
Cre10.g419900			C			
Cre10.g420350	PSAE	Photosystem I 8.1 kDa reaction center subunit IV	C, CP	G	Cr	
Cre10.g420537		Sphingomyelin phosphodiesterase 2	O			
Cre10.g421150		Glycosyltransferase 14 Family Member	C			
Cre10.g427950		Leucine-rich repeat-containing protein	O			
Cre10.g429400	MCG1	FAST Leu-rich domain-containing, stabilize petG mRNA	O		Cr	
Cre10.g429601		Cell death-related nuclease 2	O			
Cre10.g431950		Dual-specificity kinase	C			
Cre10.g433350		Squamosa promoter-binding-like protein 10-related	O			
Cre10.g433900		E3 ubiquitin-protein ligase HUWE1 (HUWE1, MULE, ARF-BP1)	O			
Cre10.g440450	PSB28	Photosystem II subunit 28	C	G		
Cre10.g445100	CGL50	Predicted protein	C	G		
Cre10.g448950		Endonuclease/Exonuclease/Phosphatase family	C		Cr	
Cre10.g452800	LCIB	Low-CO_2_-inducible protein	C, CP		Cr	
Cre10.g457900			O			
Cre10.g466500	CPL12	Glyoxylase family protein (yaeR)	C	G	Cr	
Cre11.g467644	CLPB1	ClpB chaperone, Hsp100 family ClpB chaperone, Hsp100 family	O			
Cre11.g467689	PETC	Rieske iron-sulfur subunit of the Cytochrome *b*_*6*_*f* complex, chloroplast precursor	C, CP	G	Cr	
Cre11.g467690		Glutathione transferase/S-(hydroxyalkyl)glutathione lyase	O			
Cre11.g467700	UPD1	Uroporphyrinogen-III decarboxylase	C, CP	G		
Cre11.g467712		Structural maintenance of chromosomes SMC family member	C		Cr	
Cre11.g469450	CGL124	Adhesion regulating molecule 1 110 kDa cell membrane glycoprotein	O	G		
Cre11.g476100			C		Cr	
Cre11.g477625	(CHLH2)	Magnesium chelatase subunit H	C		Zm	
Cre12.g483650		Serine/Threonine-protein kinase STN7, chloroplastic	O, CP			
Cre12.g486750			C			
Cre12.g487500	CGL61, NYE1	Stay green 1 protein, predicted protein	C			
Cre12.g494000	CGL82	Predicted protein/BRCA1-associated protein	O	G		
Cre12.g494350		Endomembrane family protein 70	O, CP			
Cre12.g494550	RNP10	RNA-binding protein	C			
Cre12.g496250			C			
Cre12.g499500	SAC3	Sulfur acclimation protein, Snf1-like Ser/Thr protein kinase	O			
Cre12.g502000	FAP253	Flagellar associated protein	O			
Cre12.g508850	GST8	Glutathione S-transferase, GST, superfamily, GST domain containing	C			
Cre12.g509001	RPK2	Mitogen-activated protein kinase	n/a		Cr	M
Cre12.g509050	PSBP3	OEE2-like protein of thylakoid lumen	C	G		M
Cre12.g510034		Tetratricopeptide repeat protein 33, Osmosis responsive factor	O			
Cre12.g510650	FBP1	Fructose-1,6-bisphosphatase	C, CP		Cr	
Cre12.g510750			C			
Cre12.g511400		Cyclin-related protein with PPR domain	O		Zm, At	
Cre12.g511650		Auxilin/cyclin G-associated kinase-related	O			
Cre12.g517681			C		Cr	M
Cre12.g517700	NYC1, SDR21	Short-chain dehydrogenase/reductase, probably chlorophyll b reductase	O	G		M
Cre12.g522000			C			
Cre12.g524250			C		Cr	
Cre12.g524300	CGL71	Tricopentapeptide repeat, Protein *O-*GlcNAc transferase	C, CP	G	Cr, Zm	
Cre12.g524500	RMT2	Rubisco small subunit N-methyltransferase	O		Cr	
Cre12.g524700		Pyrimidine and pyridine-specific 5’-nucleotidase (SDT1)	O		Zm	
Cre12.g527600		Polyglutamine-binding protein 1 (PQBP1, NPW38)	O			
Cre12.g528250		WASP-interacting protein VRP1/WIP, contains WH2 domain	O			
Cre12.g531050	RAA3	PsaA mRNA maturation factor 3	C		Cr	
Cre12.g538650	HEM4	Uroporphyrinogen-III synthase	C			
Cre12.g543100		tRNA (adenine-N(1)-)-methyltransferase non-catalytic subunit (TRM6, GCD10)	O			
Cre12.g549050	STR1	Strictosidine synthase	O			
Cre12.g549500		Pyrimidodiazepine synthase	C			
Cre12.g554800	PRK1	Phosphoribulokinase	C, CP	G	Cr	
Cre12.g559050		BCDNA, fatty acid metabolism, transport	O			
Cre13.g563150	CGLD8	Predicted protein	C, CP	G	Zm	
Cre13.g569700			C		Cr	
Cre13.g573000		Ribulose-1,5-bisphosphate carboxylase/oxygenase small subunit N-methyltransferase I-related	C			
Cre13.g574150		L-2-hydroxyglutarate dehydrogenase / L-alpha-hydroxyglutarate dehydrogenase (FAO10)	C			
Cre13.g574200	PAP2	Poly(A) polymerase/Topoisomerase related protein	C			
Cre13.g575000	CCS1	Protein required for Cytochrome *c* synthesis/biogenesis, chloroplastic	O	G	Zm	
Cre13.g578750	TBA1	PsbA translation factor	C, CP			
Cre13.g579450	CST1	Chlamydomonas-specific membrane transporter of unknown function	O			
Cre13.g580650		Serine/Threonine-protein phosphatase 2A activator (PPP2R4, PTPA)	C			
Cre13.g580850		Chloroplast 50S ribosomal protein L22-related	C			
Cre13.g584350			O			
Cre13.g584950			C			
Cre13.g586750		Transportin 3 and Importin 13	O		Cr	
Cre13.g605650		Betaine aldehyde dehydrogenase/oxidase	O			
Cre13.g607000		Cytosol nonspecific dipeptidase/Prolylglycine dipeptidase	O			
Cre14.g608652			O			
Cre14.g616600		Dynamin or thiamine synthase (FZL)	C		Cr	
Cre14.g621650		Malonyl-CoA acyl carrier protein transacylase (fabD)	C, CP			
Cre14.g624350	VTE6	MPBQ/MSBQ methyltransferase	C, CP			
Cre15.g635450			O			
Cre16.g656000		Sphingomyelin phosphodiesterase 2	O			
Cre16.g658950			C		Cr	
Cre16.g661250		Thioredoxin peroxidase	O			
Cre16.g662150	CCB1, CPLD51	CPLD51 protein, required for Cyt *b*_*6*_ assembly	C			
Cre16.g663050		Guanylate-binding family protein	O			
Cre16.g663600		MFS transporter, ACS family, solute carrier family 17 (sodium-dependent inorganic phosphate cotransporter)	O			
Cre16.g665250	APE1	Thylakoid associated protein required for photosynthetic acclimation to variable light intensity	C, CP	G		
Cre16.g665400		Small nuclear ribonucleoprotein SmD1	O, CP			
Cre16.g665800	SSS4	Soluble starch synthase	C			M
Cre16.g666050	CPLD49, SCD1	Saccharopine dehydrogenase	C	G	Cr	M
Cre16.g666150	ODA1	Flagellar outer dynein arm-docking complex protein 2	O			
Cre16.g668700			O		Cr	
Cre16.g670754		Voltage and ligand gated potassium channel	C			
Cre16.g675100	CrCO	Zinc finger protein CONSTANS-related	C	G		
Cre16.g677050		Adenylate and guanylate cyclase catalytic domain//Bacterial extracellular solute-binding protein	C			
Cre16.g678808		U4/U6 small nuclear ribonucleoprotein Prp4 (contains WD40 repeats)	O			
Cre16.g679950	RFC3	DNA replication factor C complex subunit 3	O			
Cre16.g682100		Tropinone reductase I	O			
Cre16.g684250			C			
Cre16.g684300		3-Hydroxyisobutyrate dehydrogenase-related	C, CP		Zm	
Cre16.g684900			C			
Cre16.g686510			C			
Cre16.g687966	FAP5	Tetratricopeptide repeat, Flagellar associated protein	C			
Cre16.g689150	SQD3	Sulfolipid synthase	C			
Cre16.g692228	MARS1	Serine/Threonine protein kinase	C		Cr	
Cre17.g702150	TRX20, HCF164	Thioredoxin-like protein HCF164, chloroplastic	C, CP	G	Cr	M
Cre17.g702500	TAB2	DUF1092, PsaB RNA binding protein	C, CP	G	Zm	M
Cre17.g704000		Polyvinyl-alcohol oxidase/PVA oxidase	C			
Cre17.g704350		Glyoxalase domain-containing protein 4	O			
Cre17.g710800	NFU3	Iron-sulfur cluster assembly protein	C, CP	G		
Cre17.g711150	FAD2	omega-6 Fatty acid desaturase (delta-12 desaturase)	O			
Cre17.g712850	TRX23	Thiol-disulfide isomerase and thioredoxin	O		Cr	
Cre17.g717400	miaA, TRIT1	tRNA dimethylallyltransferase	O	G		
Cre17.g719450		Ca2+/calmodulin-dependent protein kinase, EF-Hand protein superfamily//Serine/threonine protein kinase	C			
Cre17.g721350	GST13	Glutathione S-transferase	O			
Cre17.g721950		E3 Ubiquitin-protein ligase ARI2-related	O			
Cre17.g722300			O			
Cre17.g724600	PAO2	Pheophorbide *a* oxygenase, Rieske iron-sulfur cluster protein	C			M
Cre17.g724700	PAO1	Pheophorbide *a* oxygenase, Rieske iron-sulfur cluster protein	C			M
Cre17.g725750	SSA2	60 kDa SS-A/Ro ribonucleoprotein	O			
Cre17.g731100	CPL14	Uncharacterized conserved protein	C	G		
Cre17.g734548	PPD2	Pyruvate phosphate dikinase, chloroplastic	C		Zm	

^1^ C, predicted to be chloroplast targeted by Predalgo or ChloroP; O, other; n/a, not analyzed; CP, found in chloroplast proteome by Terashima et al, (2011).

^2^ G, GreenCut2.

^3^ Identified in other photosynthesis mutant library studies Chlamydomonas (Cr), Maize (Zm), Arabidopsis (At).

^4^ M, Multiple strong candidates in this mutant. See S4 Table for further detail.

## Discussion

We successfully used high-throughput, low-coverage WGS for the identification of plasmid insertion sites in our *Chlamydomonas* photosynthesis mutant collection (ARC). This approach has a much higher efficiency than PCR-based FST isolation. From the larger collection of 2800 mutants [7] from which ARC was derived, we recovered FSTs from only 17% of the mutants, whereas our WGS identified insertions in 509 out of 581 non-redundant ARC mutants (88% success among the population). We attribute this improvement to the fact that insertion site identification by WGS is not dependent on the intactness or sequence continuity of the inserted plasmid sequence, and therefore WGS overcomes complications such as plasmid concatemerization and loss of plasmid ends to which PCR primers need to anneal. Most importantly, it completely bypasses the need for PCR from the GC- and repeat-rich genome of *Chlamydomonas*. Even with relatively low average WGS coverage (~7x), we also identified 68 deletions that were not associated with plasmid insertions, some of which may be causative mutations for photosynthesis-related phenotypes that are unlinked to the plasmid insertion in specific mutants.

A previous study using WGS to identify DNA insertion events in *Chlamydomonas* [21] provides the most direct comparison with our results. Lin et al. (2018) analyzed paromomycin-resistant insertional mutants derived from electroporation instead of the glass bead transformation method that we used to generate either paromomycin- or zeocin-resistant mutants [9]. They sequenced 20 transformants in 10 pools of two strains and verified 38 insertions, obtaining an average of 1.9 insertions per strain. In contrast, we found a total of 554 insertions in 509 mutants, resulting in a lower average of ~1.1 insertions per mutant. Lin et al. (2018) found that more than half (11 of 20) of their strains had more than one insertion event, and a larger collection of 1935 mutants derived from electroporation exhibited multiple insertions in 26% of strains [10]. We found multiple insertions in 8% (43 out of 509) of the ARC mutants, suggesting that glass bead transformation of *Chlamydomonas* results in a higher frequency of single-copy insertions. Lin et al. (2018) identified one-sided insertions in ~40% of their mutants, whereas we observed only ~4% (21 out of 554 insertion events), despite the lower average WGS coverage in our study (~7x vs. ~15x). The frequency of complex rearrangements in our study (19%) was comparable to that observed by Lin et al. (25%), however, as previously noted by us and others [7,10,21,48], glass bead transformation seems to be frequently associated with larger deletions of genomic DNA at the sites of DNA insertion than electroporation, a finding that was clearly evident in our WGS data (Fig 3A).

In part because of the occurrence of larger deletions, 1470 genes were disrupted in 509 ARC mutants. As expected, this list is enriched for genes that encode proteins with annotated functions in photosynthesis and tetrapyrrole synthesis, and it includes 78 GreenCut2 genes [31]. We examined the affected genes in each mutant to identify possible causative genes using several criteria, including GreenCut2 membership, existence of protein domains suggestive of a function in photosynthesis, and occurrence of multiple mutant alleles in the ARC. We also searched for overlaps with available photosynthesis mutant datasets, namely CLiP (*Chlamydomonas*), PML (maize), DEPI (*Arabidopsis*), and those found co-expressed with photosynthesis genes (*Chlamydomonas*). The CLiP collection has been used to identify mutants that are defective in photosynthetic growth in pooled cultures [9]. This study identified 303 candidate photosynthesis genes. We identified 43 of those 303 genes in our list of 253 higher-confidence genes (Table 2 and S4 Table). The maize PML consists of approximately 2100 photosynthesis mutants that contain 50 to 100 *Mu* transposable elements per individual. It is estimated to be a saturated collection with 3–4 mutant alleles for ~600 genes [43]. The FSTs of this library were obtained with Illumina sequencing of fragmented gDNA that was enriched for the *Mu* element [22]. Our higher-confidence candidate gene list overlapped with 17 genes identified from the maize PML (http://pml.uoregon.edu/photosyntheticml.html). DEPI screening of 300 *Arabidopsis* mutants affecting genes that encode chloroplast-targeted proteins (Chloroplast 2010 project, http://www.plastid.msu.edu/) identified 12 mutants with altered photosynthetic response [44]. These mutants likely represent disruption in genes that are conditionally important in acclimation to changing light environments. Two of the 12 genes found through DEPI overlapped with our higher-confidence photosynthesis candidate gene list. The largest overlap (83 genes) was observed between our higher-confidence list and the group of photosynthesis-related genes defined based on co-expression analysis [42].

For two of the higher-confidence photosynthesis genes, *LPA3* and *PSBP4*, we validated the insertion-associated lesions for four of the ARC mutants and demonstrated their requirement for photoautotrophic growth (Fig 5). LPA3 is a GreenCut2 protein (CPLD28) that contains a DUF1995 domain. Insertion mutants containing large or small deletions in *LPA3* (Cre03.g184550) were acetate-requiring and exhibited a severe defect in PSII function even in the dark, as evidenced by F_v_/F_m_ values near zero (Fig 5). A mutant (CAL014_01_01) affecting Cre02.g105650, which was recently identified as the *Chlamydomonas* ortholog of *Arabidopsis LPA2* [49], was also found to require acetate and was highly light-sensitive. The phenotypes of these *Chlamydomonas* mutants are much more severe than those of *Arabidopsis lpa2* and *lpa3*, indicating that *Chlamydomonas* is more dependent on these proteins for PSII assembly. There are two additional genes encoding DUF1995 proteins in the *Chlamydomonas* genome, Cre06.g281800 and Cre08.g369000. The mutant CAL038_02_36 is disrupted in Cre06.g281800. It does not grow photoautotrophically but is able to grow in LL and HL in the presence of acetate. Interestingly, this mutant also has an F_v_/F_m_ of nearly zero in the dark (S1 Table), similar to the *lpa3-1* (Fig 5C) and *lpa3-2* (S1 Table) mutant alleles. The severe phenotypes of these mutants in *Chlamydomonas* indicate non-overlapping functions in PSII assembly of the gene products of *LPA2*, *LPA3*, and Cre06.g281800.

PSBP (encoded by *PSBP1*/*OEE2* in *Chlamydomonas*) together with PSBO and PSBQ constitute the oxygen-evolving complex (OEC) of PSII [50,51]. In green algae and plants, PSBP appears to have expanded into a large family of proteins sharing similar domains beyond the canonical PSBP of the OEC. The *Chlamydomonas* genome contains 13 additional genes encoding proteins with PsbP-like domains whose individual functions are unknown. We showed that PSBP4 is required for photoautotrophic growth in *Chlamydomonas*, ruling out redundancy in its function with other PSBP-like domain-containing proteins. An *Arabidopsis* ortholog of CrPSBP4 (AT4G15510, PPD1) has been shown to play a role in PSI assembly [40,41], which is consistent with the light-sensitivity and the lack of PSI subunits accumulation of our *psbp4-1* mutant (Fig 5). Two other members of the PSBP family, *PSBP3*, and *PSBP9*, were found to be disrupted in the ARC. The large family of PSBP-like domain-containing proteins is speculated to have resulted in divergence of their functions [52], and the availability of mutants in these genes should help to reveal their functions.

Of the 253 higher-confidence candidate photosynthesis genes that we curated based on WGS analysis of the ARC, only 70 have a previously demonstrated function in photosynthesis. This is similar to the results of pooled growth analysis of ~60,000 *Chlamydomonas* insertional mutants by Li et al. (2019), which revealed 303 candidate photosynthesis genes, of which only 65 have previously known roles in photosynthesis [9]. Thus, 238 genes in the study of Li et al. (2019) and 183 genes in our study remain to be analyzed experimentally to determine their specific functions in photosynthesis. Only 43 genes are shared by these two sets of candidate photosynthesis genes, and yet this is a statistically significant overlap (Fisher’s exact test rejects the null hypothesis with a P-value of 0.05). This overlap is lower than might be expected but is likely due to the fact that both the CLiP and ARC mutant collections are based on a total of ~62,000 and ~49,000 insertional mutants, respectively, which is not sufficient to saturate the *Chlamydomonas* genome for mutations affecting photosynthesis (assuming a Poisson distribution, an average exon length per gene of 1,583 bp [15], and a total genome size of 111 Mb (https://phytozome.jgi.doe.gov/pz/portal.html#!info?alias=Org_Creinhardtii), the likelihood of a library of 60,000 insertional mutants hitting a gene is 58%). In addition, the presence of incomplete knock-outs in the libraries may be contributing, which is a challenge for all studies of this type in eukaryotic genomes containing non-coding regions (~50% of CLiP library insertions were in exons, Fig 2A in Li et al). Another possible reason is the difference in the experimental designs of the studies. The CLiP mutant pool was selected in minimal medium at 500 μmol photons m^-2^ s^-1^ in 20-L liquid cultures that would have experienced self-shading [9]. In contrast, ARC mutants were screened for even relatively small differences in photoautotrophic growth under more severe HL conditions on plates rather than in liquid medium, which might have led to identification of a different set of genes. Nonetheless, these studies suggest that there are still many more photosynthesis genes that remain to be identified, which highlights the enormous potential for future validation and discovery of new proteins involved in oxygenic photosynthesis.

## Material and methods

### Strains and culture conditions

Mutants described in this work were generated from wild-type strain 4A+ (CC-4051) in the 137c background. Cells were grown mixotrophically (ac) on Tris-acetate-phosphate (TAP) medium and photoautotrophically (min) on minimal high-salt medium (HS) medium [53] in low light (LL) of 60–80 μmol photons m^-2^ s^-1^ and high light (HL) of 350–400 μmol photons m^-2^ s^-1^. LL and HL conditions were obtained using GE F25T8/SPX41/ECO and Sylvania F72T12/CW/VHO fluorescent bulbs, respectively.

### Generation of mutant library by insertional mutagenesis

Detailed methods used to generate the mutants analyzed in this work were previously described [7,26]. In brief, cells growing in log phase were harvested and transformed with linearized plasmid DNA following the glass beads method [54]. Either 600 ng (pMS188 and pBC1) or 1 μg (pSP124S) of DNA was used for 51x10^6^ cells in 300 μL, under which conditions ~70% of the mutants generated contained a single insertion [26].

### Genomic DNA preparation and whole-genome sequencing

*Chlamydomonas* cultures were grown in 20 mL TAP to stationary phase, and genomic DNA was extracted using an alkaline lysis buffer (50 mM Tris-HCl (pH 8), 200 mM NaCl, 20 mM EDTA, 2% SDS, 1% PVP 40,000, 1 mg/mL Proteinase K) followed by phenol-chloroform extraction. DNA was collected, washed and eluted using DNeasy Plant mini-columns (QIAGEN). The resulting quality of the DNA was confirmed to be A_260_/A_280_ of approximately 1.8 and A_260_/A_230_ of >2. Plate-based DNA library preparation for Illumina sequencing was performed on the PerkinElmer Sciclone NGS robotic liquid handling system using Kapa Biosystems library preparation kit. 200 ng of sample DNA was sheared to 600 bp using a Covaris LE220 focused ultrasonicator. The sheared DNA fragments were size selected by double-SPRI, and then the selected fragments were end-repaired, A-tailed, and ligated with Illumina-compatible sequencing adaptors from IDT containing a unique molecular index barcode for each sample library. The prepared libraries were quantified using Kapa Biosystem’s next-generation sequencing library qPCR kit and run on a Roche LightCycler 480 real-time PCR instrument. The quantified libraries were then multiplexed with other libraries, and the pool of libraries was then prepared for sequencing on the Illumina HiSeq sequencing platform utilizing a TruSeq paired-end cluster kit, v4, and Illumina’s cBot instrument to generate a clustered flow cell for sequencing. Sequencing of the flow cell was performed on the Illumina HiSeq2500 sequencer using HiSeq TruSeq SBS sequencing kits, v4, following a 2x150 indexed run recipe. The reads were aligned to the reference genome using BWA-mem. To identify plasmid insertion sites, discordant paired-end reads with one end mapping to the plasmid used for mutagenesis and the other to a chromosome location were mapped and manually validated for each mutant using Integrated Genome Viewer (IGV 2.3.94) (http://software.broadinstitute.org/software/igv/home). Putative structural variations unpaired to the plasmid sequence were called using a combination of BreakDancer (filtered to quality 90+) and Pindel and manually validated using IGV. Resulting genome sequences of 79 mutants were not unique (33 were duplicated, three were triplicated and one was quadruplicated). In all cases the mutants sharing similar sequences came from the same agar plate and sequencing plate, suggesting that it could be due to an error at the genome extraction step or in maintenance of the mutant strains; these mutants were not included in further analysis. All sequence files are available from the NCBI SRA database (https://www.ncbi.nlm.nih.gov/sra). The SRA accession numbers for each of the mutants are listed in S1 Appendix.

### Molecular analyses of mutants by PCR and mutant complementation

Deletions predicted from genome sequences were confirmed by using PCR primers that anneal proximal to the borders and within the deletions. The insertion of the plasmid sequence accompanied by a 4 bp-deletion in *lpa3-3* was sequenced from the PCR product from the predicted region. Primers used for PCRs indicated in Fig 4 are listed in S5 Table. For complementation of *lpa3-1*, *lpa3-2*, and *lpa3-3*, a 3531 bp genomic fragment containing the full length *CrLPA3* gene (Cre03.g184550) with 1209 bp upstream of the start codon and 719 bp downstream of the stop codon was amplified using primers Comp11F and Comp11R. This fragment was subsequently Gibson cloned into the vector pSP124S using primers PS1362 and PS1363 to inverse PCR around pSP124S. For complementation of mutant *psbp4-1*, a 3246 bp genomic fragment containing the full length *CrPSBP4* gene (Cre08.g362900), including 1209 bp upstream of the start codon and 719 bp downstream of the stop codon, was amplified using primers Comp12F and Comp12R and similarly cloned into vector pSP124S. Primer sequences are listed in S4 Table. Constructs for complementation were transformed into the respective mutants using the glass bead method [54]. Colonies were selected on 10 μM zeocin TAP agar plates and screened for rescued individuals by measuring F_v_/F_m_ as described below.

### F_v_/F_m_ measurement

*Chlamydomonas* strains were grown on agar plates in Dark+ac, LL-min, or HL-min, and F_v_/F_m_ (F_m_-F_o_/F_m_) was measured using a chlorophyll fluorescence video imager (IMAG-MAX/L, WALZ). Plates with the streaks of strains were dark-acclimated for 30 min and exposed to a pulse of saturating light (4000 μmol photons m^-2^ s^-1^). Fluorescence images of F_m_ and F_o_ were captured during saturating pulses, and false-color images of F_v_/F_m_ were generated.

### Immunoblot analysis of photosystem subunits

WT and mutants were grown in TAP to early log phase under very low light (0~2 μmol photons m^-2^ s^-1^). Total protein was solubilized as previously described [29] and extracted using the methanol/chloroform method [55]. Each sample was quantified for protein concentration using BCA protein assay (Pierce) to load 5 μg protein in each lane of pre-cast SDS-PAGE Any kD Mini-PROTEAN gels (Bio-Rad), except for the dilutions of the WT samples. Proteins were transferred from the gel to a polyvinylidene difluoride membrane (Immobilon-FL 0.45 μm, Millipore) via wet-transfer for immunodetection. The primary polyclonal antibodies raised against AtpB (AS16 3976), D1 DE-loop (AS10 704), CP43 (AS11 1787), PsaA (AS06 172), and PsaD (AS09 461) were obtained from Agrisera. Donkey anti-rabbit IgG antibody with horseradish peroxidase (1:10,000; GE Healthcare) was used as a secondary antibody and visualized using SuperSignal West Femto Maximum Sensitivity Substrate (Thermo Scientific) chemiluminescent system on a ChemiDoc MP imaging system (Bio-Rad).

## Supporting information

S1 Fig Proportion of different types of insertions observed in ARC. The frequency of the different types of insertions compared to the total number of insertions observed in the mutant library. Some insertions coexist with another insertion in a mutant. The number of mutants grouped by the types of insertions it contains is listed along with the number of insertions accounted for in that group.
(PDF)

S2 Fig Genetic linkage test of par^R^ and *ac*^-^ phenotypes. Mutants (*ac*^-^ par^R^) were crossed with WT (*AC*^*+*^ par^S^) cells. For a mutant with a single insertion that causes *ac*^-^ par^R^, the two phenotypes segregate 2:2 in the resulting progeny. Each colony in the images arose from a single zygospore that was a mix of four genotypes (tetrad progenies). In the left column, half of the progenies from a zygospore grew since a cross between a single allele of *ac*^-^ and WT will result in half of the progeny being *AC*^*+*^. In the right column, progenies were selected on paromomycin and on minimal media. If the insertion of par^R^ at a given locus results in the *ac*^-^ phenotype (i.e., genetically linked), none of the progenies grow on minimal media as in the upper four mutants. In the lower two mutants, growth was observed because the *ac*^-^ and par^R^ genotypes are separate mutations at distinct loci that segregated in some of the progenies.
(PDF)

S3 Fig Two mutant alleles in tocopherol cyclase (Cre01.g013801, *VTE1*) in ARC. Schematic representation of the disruption sites in CAL014_01_19, a strictly acetate-requiring mutant and CAL032_02_19, a mutant with comparatively moderate phenotype.
(PDF)

S1 Table Plasmid-paired and unpaired discordant sites detected in ARC by WGS and mutant phenotypes. (XLSX)

S2 Table Mutants with deletions unassociated with plasmid insertion. (XLSX)

S3 Table Total genes affected in ARC and their description. (XLSX)

S4 Table Higher-confidence candidate genes and corresponding mutants. (XLTX)

S5 Table List of PCR primers used in this study. (XLSX)

S1 Appendix SRA accession IDs. (XLSX)

S2 Appendix Numerical data for histograms and graphs. (PDF)
