# Supplementary material for: Discovery of photosynthesis genes through whole-genome sequencing of acetate-requiring mutants of Chlamydomonas reinhardtii
Source: PLoS Genet. 2021 Sep 7;17(9):e1009725. doi: 10.1371/journal.pgen.1009725 (PMC8448359; doi:10.1371/journal.pgen.1009725)
Supplement: S1 Fig — The frequency of the different types of insertions compared to the total number of insertions observed in the mutant library. Some insertions coexist with another insertion in a mutant. The number of mutants grouped by the types of insertions it contains is listed along with the number of insertions accounted for in that group. (PDF) [file pgen.1009725.s001.pdf]

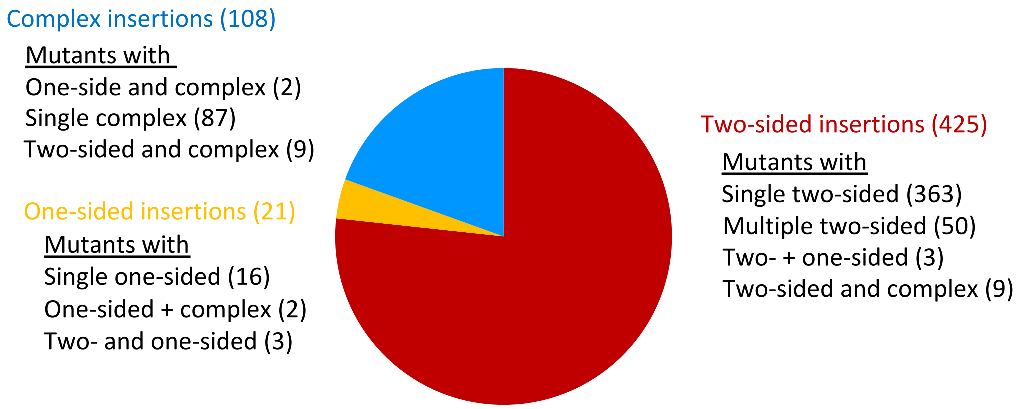

S1 Fig. Proportion of different types of insertions observed in ARC.
The frequency of the different types of insertions compared to the total number of insertions observed in the mutant library. Some insertions coexist with another insertion in a mutant. The number of mutants grouped by the types of insertions it contains is listed along with the number of insertions accounted for in that group.
