# Supplementary material for: Discovery of photosynthesis genes through whole-genome sequencing of acetate-requiring mutants of Chlamydomonas reinhardtii
Source: PLoS Genet. 2021 Sep 7;17(9):e1009725. doi: 10.1371/journal.pgen.1009725 (PMC8448359; doi:10.1371/journal.pgen.1009725)
Supplement: S2 Fig — Mutants (ac- parR) were crossed with WT (AC+ parS) cells. For a mutant with a single insertion that causes ac- parR, the two phenotypes segregate 2:2 in the resulting progeny. Each colony in the images arose from a single zygospore that was a mix of four genotypes (tetrad progenies). In the left column, half of the progenies from a zygospore grew since a cross between a single allele of ac- and WT will result in half of the progeny being AC+. In the right column, progenies were selected on paromomycin and on minimal media. If the insertion of parR at a given locus results in the ac- phenotype (i.e., genetically linked), none of the progenies grow on minimal media as in the upper four mutants. In the lower two mutants, growth was observed because the ac- and parR genotypes are separate mutations at distinct loci that segregated in some of the progenies. (PDF) [file pgen.1009725.s002.pdf]

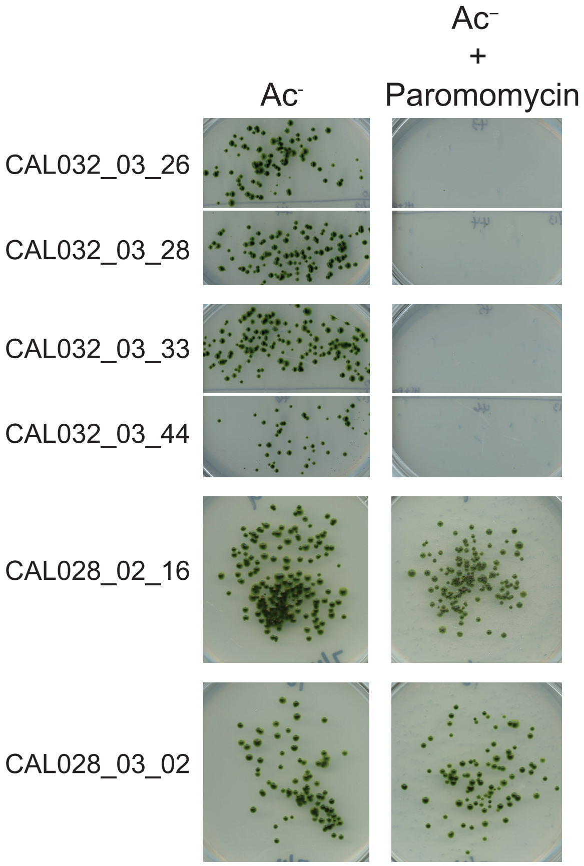

S2 Fig. Genetic linkage test of par^R and *ac*$^-$ phenotypes.
Mutants (*ac*$^-$ *par*^{R}) were crossed with WT (*AC*$^+$ *par*^{S}) cells.
For a mutant with a single insertion that causes *ac*$^-$ *par*^{R}, the two phenotypes segregate 2:2 in the resulting progeny. Each colony in the images arose from a single zygospore that was a mix of four genotypes (tetrad progenies). In the left column half of the progenies from a zygospore grew since a cross between a single allele of *ac*$^-$ and WT will result in half of the progeny being *AC*$^+$. In the right column, progenies were selected on paromomycin and on minimal media. If the insertion of *par*^{R} at a given locus results in the *ac*$^-$ phenotype (i.e., genetically linked), none of the progenies grow on minimal media as in the upper four mutants. In the lower two mutants, growth was observed because the *ac*$^-$ and *par*^{R} genotypes are separate mutations at distinct loci that segregated in some of the progenies.
