# Supplementary material for: Discovery of photosynthesis genes through whole-genome sequencing of acetate-requiring mutants of Chlamydomonas reinhardtii
Source: PLoS Genet. 2021 Sep 7;17(9):e1009725. doi: 10.1371/journal.pgen.1009725 (PMC8448359; doi:10.1371/journal.pgen.1009725)
Supplement: S3 Fig — Schematic representation of the disruption sites in CAL014_01_19, a strictly acetate-requiring mutant and CAL032_02_19, a mutant with comparatively moderate phenotype. (PDF) [file pgen.1009725.s003.pdf]

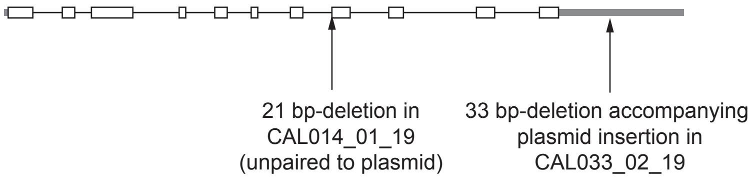

S3 Fig. Two mutant alleles in tocopherol cyclase (Cre01.g013801, *VTE1*) in ARC. Schematic representation of the disruption sites in CAL014_01_19 a strictly acetate-requiring mutant and CAL032_02_19, a mutant with comparatively moderate phenotype.
