# Supplementary material for: Discovery of photosynthesis genes through whole-genome sequencing of acetate-requiring mutants of Chlamydomonas reinhardtii
Source: PLoS Genet. 2021 Sep 7;17(9):e1009725. doi: 10.1371/journal.pgen.1009725 (PMC8448359; doi:10.1371/journal.pgen.1009725)
Supplement: S2 Appendix — (PDF) [file pgen.1009725.s010.pdf]

S2 Appendix

Numerical data for graphs

Fig. 2 Piechart

Single two-sided insertion	361
Multiple two-sided insertions	24
One-sided insertion and a two-sided insertion	3
Single one-sided insertion	16
One-sided and complex insertions	2
Single complex insertion	87
Two complex insertions	9
Two-sided insertion and complex insertion	7

Fig. 3A Histogram

	Number of mutants
Dup	29
Perfect	22
del 1-10	43
del 11-100	88
del 101-1000	44
del 1001-10,000	36
del 10,001-100,000	157
del 100,001-	2

Fig. 3B Histogram

Number of genes affected	Number of mutants
0	23
1	216
2	20
3	21
4	22
5	39
6	16
7	14
8	13
9	7
10	8
11	10
12	3
13	2
14	3

15	2
25	1
29	1

Fig. 4A Histogram

Number of mutant alleles	Number of genes
1	1053
2	212
3	94
4	17
5	14
6	10
7	6
8	1

Fig. 5C Graph

	TAP			HS		TAP			HS	
	Dark	LL	HL	LL	HL	Dark	LL	HL	LL	HL
	average	average	average	average	average	Stdev	Stdev	Stdev	Stdev	Stdev
4a	0.456	0.653	0.516	0.570	0.479	0.013	0.009	0.013	0.020	0.008
lpa3-2	0.070	0.070	0.040	0.032	0.000	0.013	0.013	0.036	0.055	0.000
comp1	0.610	0.610	0.330	0.620	0.517	0.030	0.030	0.091	0.022	0.008
comp2	0.612	0.612	0.504	0.588	0.511	0.004	0.004	0.024	0.015	0.025
psbp4-1	0.508	0.072	0.000	0.114	0.000	0.020	0.029	0.000	0.012	0.000
comp1	0.634	0.622	0.473	0.645	0.489	0.007	0.006	0.038	0.012	0.009
comp2	0.610	0.617	0.538	0.644	0.482	0.013	0.016	0.019	0.004	0.010
